# Enteric duplication cysts in children: varied presentations, varied imaging findings

**DOI:** 10.1007/s13244-018-0660-z

**Published:** 2018-10-11

**Authors:** Cinta Sangüesa Nebot, Roberto Llorens Salvador, Elena Carazo Palacios, Sara Picó Aliaga, Vicente Ibañez Pradas

**Affiliations:** 1 Radiology Department, Paediatric Imaging Section, Hospital Universitario y Politécnico La Fe, Avenida Fernando Abril Martorell 106, 46026 Valencia, Spain; 2 Paediatric Surgery Department, Hospital Universitario y Politécnico La Fe, Valencia, Spain

**Keywords:** Cyst, Gastrointestinal tract, Children, Ultrasound, Magnetic resonance

## Abstract

**Abstract:**

Enteric duplication cysts (EDCs) are rare congenital malformations formed during the embryonic development of the digestive tract. They are usually detected prenatally or in the first years of life. The size, location, type, mucosal pattern and presence of complications produce a varied clinical presentation and different imaging findings. Ultrasonography (US) is the most used imaging method for diagnosis. Magnetic resonance (MR) and computed tomography (CT) are less frequently used, but can be helpful in cases of difficult surgical approach. Conservative surgery is the treatment of choice. Pathology confirms the intestinal origin of the cyst, showing a layer of smooth muscle in the wall and an epithelial lining inside, resembling some part of the gastrointestinal tract (GT). We review the different forms of presentation of the EDCs, showing both the typical and atypical imaging findings with the different imaging techniques. We correlate the imaging findings with the surgical results and the final pathological features.

**Teaching Points:**

• *EDCs are rare congenital anomalies from the digestive tract with uncertain pathogenesis.*

• *More frequently, diagnosis is antenatal, with most EDCs occurring in the distal ileum.*

• *Ultrasonography is the method of choice for diagnosis of EDCs.*

• *Complicated EDCs can show atypical imaging findings.*

• *Surgery is necessary to avoid complications.*

## Introduction

Enteric duplication cysts (EDCs) are rare congenital anomalies found anywhere along the gastrointestinal tract (GT) from the mouth to the rectum; most commonly in the ileum (33%), followed by the oesophagus (20%), colon (13%), jejunum (10%), stomach (7%) and duodenum (5%) [1–4].

The incidence is 1:4,500 births, found in 0.2% of all children, with a slight male predominance [3, 5–7].

EDCs are believed to occur between the 4th and 8th weeks of embryonic development. Their aetiology is still unknown; several theories have been proposed to explain their pathophysiology, but no single hypothesis can justify all duplications, locations and associated anomalies. Split notochord theory is often postulated [8]. The luminal recanalisation theory explains duplications in those portions of the GT that have a solid stage, including the oesophagus, small bowel and colon; nevertheless, it does not explain duplications at other levels. Incomplete or partial twinning theory could explain the colorectal duplications that are associated with duplication of genital and urinary structures. Persistent embryonic diverticula theory suggests that small diverticula, usually transient along the antimesenteric border of the intestinal wall, persist and develop intestinal duplications, although most ECDs are in the mesenteric border. The intrauterine vascular accident theory suggests that gastrointestinal duplications arise from an intrauterine vascular accident during early fetal development and may be a valid explanation for isolated duplication. These different theories lead to think that the origin of EDCs can be multifactorial [1, 2, 4, 9, 10].

Associated anomalies such as spinal defects, cardiac or urinary malformations, are reported with an incidence rate of 16–26%. Other digestive anomalies are present in about 10% of cases. Therefore, once an EDC is found, a search for other anomalies is needed [6, 10–12].

**Fig. 1:**
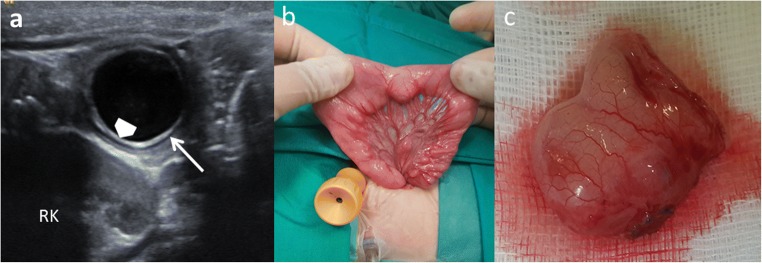
An 11-month-old boy with abdominal pain is studied. **a** US view showing the typical US features of an EDC: an inner hyperechoic epithelial lining containing the mucosa of the alimentary tract (*wide arrow*) and the outer hypoechoic layer of smooth muscle (*white long arrow*), closely attached to the gastrointestinal tract by sharing a common wall. *RK* right kidney. **b** Surgical findings: typical ileal EDC. **c** Detailed picture of the EDC after resection from the ileal wall

**Fig. 2:**
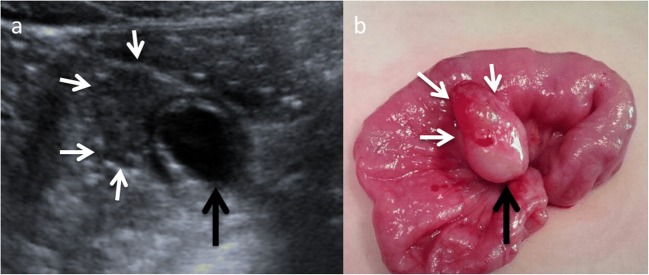
A 4-year-old boy in a routinary US control of a horseshoe kidney. **a** Abdominal ultrasound view of an EDC (*black arrow*) with a peripheral eccentric hypoechoic cap (*white arrows*). **b** Surgical findings: the gastric mucosa was visible as a polypoid mass (*white arrows*) arising from the external surface of the EDC (*black arrow*)

EDCs must have three characteristics: an epithelial lining containing the mucosa of the alimentary tract, an envelope of smooth muscle, and the cyst must be closely attached to the GT by sharing a common wall (Fig. 1). The mucosal lining does not always correlate with the adjacent gastrointestinal tissue, but the duplications are named according to the part of the GT to which these are intimately attached. Ectopic gastric mucosa is found in 20–30% of these cysts, more frequently in oesophageal and small bowel duplications [13, 14]. Prominent gastric mucosa can also be seen as a polypoid mass covering the base of the cyst, being transmural (Fig. 2) [6]. Ectopic pancreatic mucosa is most common in gastric duplications [2].

**Fig. 3:**
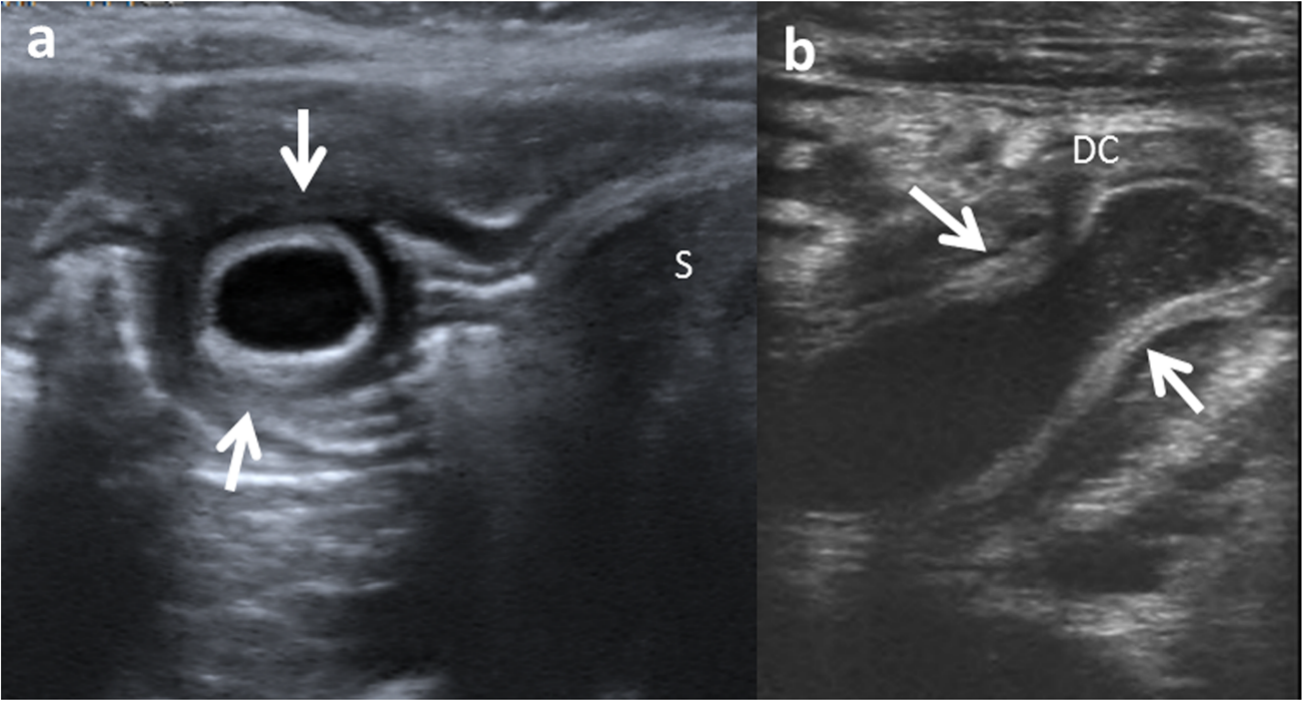
Types of duplication cysts seen in the abdominal US. **a** Spherical EDC in gastric antrum (*arrows*). *S* stomach. **b** Tubular EDC (*arrows*) next to the descending colon (*DC*)

Structurally, EDCs can be either cystic or tubular. Spherical cysts are the most common duplications (80%) and typically do not communicate with the adjacent lumen. Tubular duplication cysts (20%) run parallel to the GT, being communicated with it (Fig. 3) [4, 12, 15, 16]. Then, when a duplication cyst is tubular, the connection with GT must be demonstrated for surgical planning [17].

**Fig. 4:**
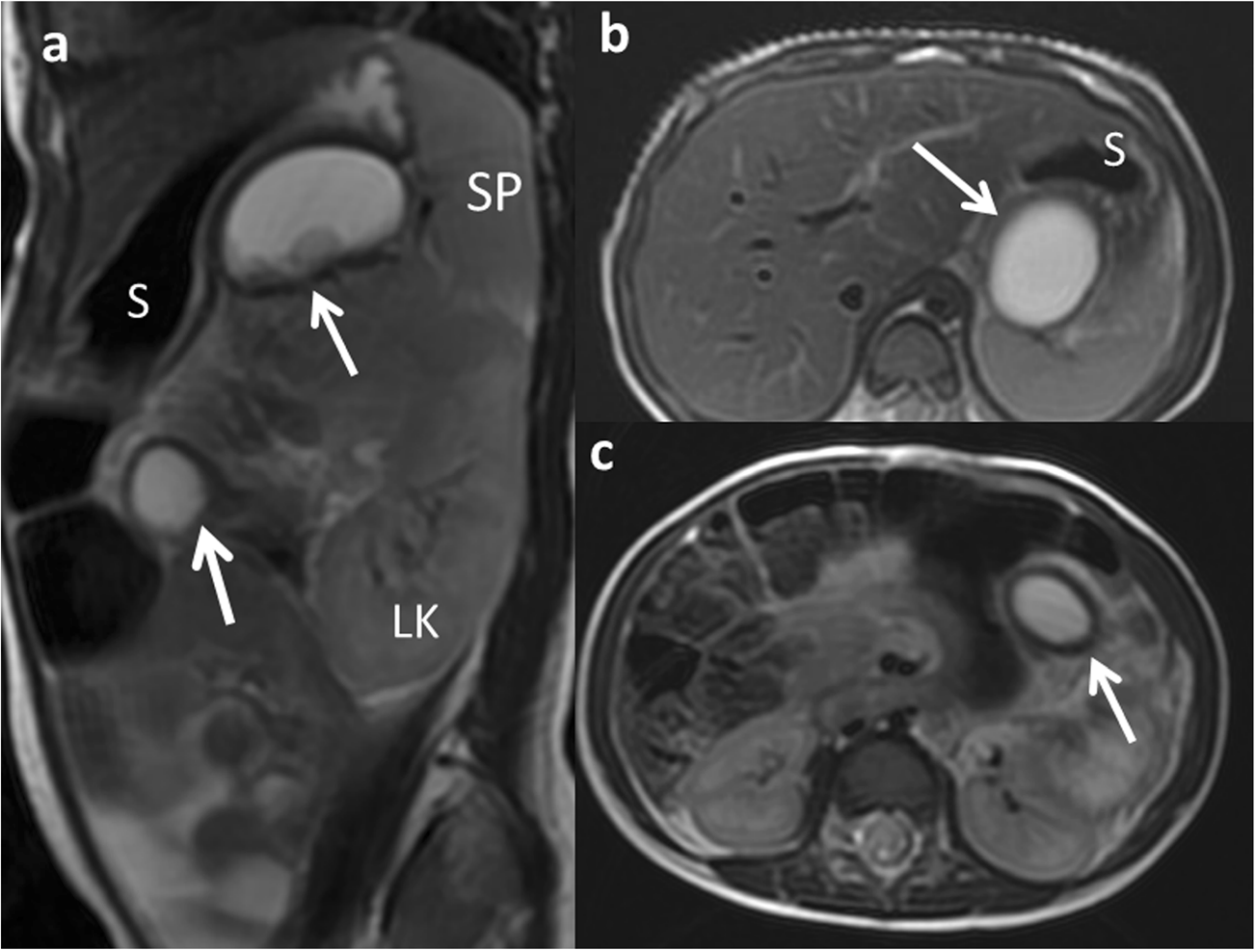
A 2-year-old girl with a splenic lesion (not shown) is studied with abdominal MR. **a** FSE T2 sagittal MRI: two similar cystic lesions are found (*arrows*). *S* stomach, *LK* left kidney, *SP* spleen. **b, c** FSE T2 axial MRI: a gastric and a jejunal duplication cyst are shown (*arrows*) in both images. Surgical findings: multiple EDCs

Multiple duplication cysts are rather uncommon (1–7%) [4, 16]. These include multiple EDCs within one segment of the GT or less frequently in two or more segments (Fig. 4) [11, 16, 18, 19].

Atypical EDC is a non-communicating isolated duplication cyst completely separated from the bowel with no communication or shared wall. A vascular insult could have led to the isolation. They are extremely rare [19, 20], especially multiple isolated EDCs, which are even rarer [19].

The size, location, type, mucosal pattern and presence of complications produce different clinical presentations and several imaging findings of the EDCs. Ultrasonography (US) is the most used imaging method for diagnosis of abdominal EDCs. Magnetic resonance (MR) and computed tomography (CT) are utilised for oesophageal EDCs and for helping in difficult surgical approaches.

We review the different forms of presentation of EDCs, showing both typical and atypical image findings with the different imaging techniques. We correlate the imaging findings with the surgical results and the final pathological features.

## Clinical presentations

**Fig. 5:**
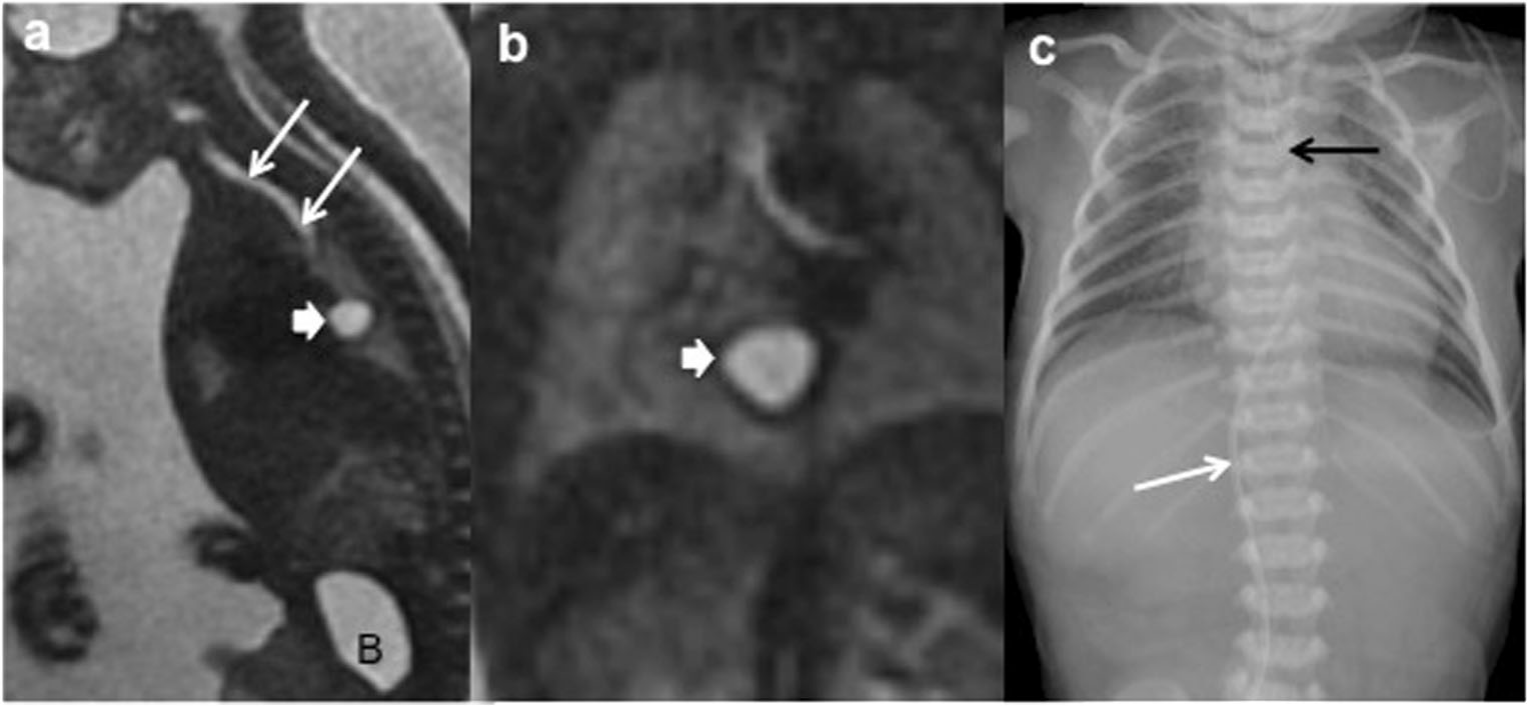
A 21-week-old fetus with polyhydramnios and absent normal gastric bubble in the US is studied. **a** Sagittal FIESTA fetal MRI showing a mediastinal cyst (thick arrow). Oesophageal atresia without fistula is suspected. *B* bladder. Trachea (*arrows*). **b** Coronal HASTE fetal MRI: detailed view of the mediastinal cyst (*thick arrow*). **c** Postnatal thoraco-abdominal radiograph: the gastric line tip is seen (*black arrow*) confirming the oesophageal atresia. The absence of air in the abdomen indicates a type-I or -II oesophageal atresia (without fistula). Venous umbilical catheter (*white arrow*)

The intrauterine presentation is increasing, mostly due to the improvement in prenatal screening US, routine second-trimester screening and improved imaging resolution. However, prenatal diagnosis of EDCs is often difficult, and US identifies only 20–30% of them, and sometimes they are discovered by chance (Fig. 5) [21, 22].

The natural history of EDCs is quite variable. The clinical presentation or onset symptoms of these malformations range from infancy and early childhood to adulthood. Almost 70% of EDCs present symptoms within the first year of life and 85% in the second one [3, 10, 14].

The signs and symptoms depend on the type and location of the duplication.

**Fig. 6:**
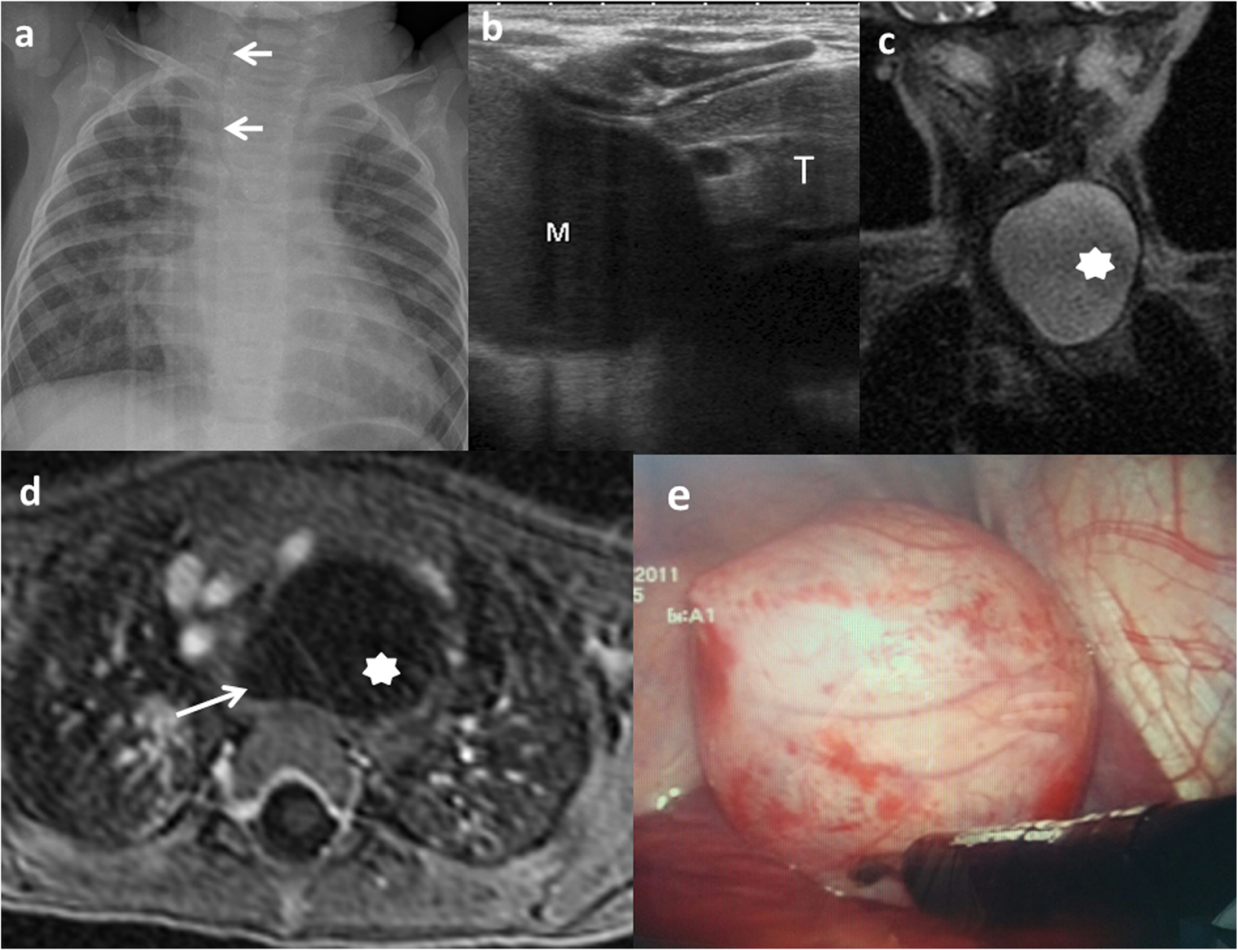
A 10-month-old boy with a congenital cardiopathy presents respiratory distress. **a** Chest X-ray: a left cervicothoracic mass is suspected displacing the trachea to the right (*arrows*). **b** Chest US: cystic mass (*M*) with slightly echogenic content inside is seen next to the thymus (*T*). Its origin is unclear. **c** Coronal view SSFSE T2 MRI: a well-delineated and hyperintense lesion (*star*) is seen. **d** Transversal view of the lesion in a gadolinium-enhanced VIBE MRI confirms the cystic nature of the mass (*star*) next to the anterior oesophageal wall (*arrow*). **e** Thoracoscopic findings: a 6 × 4-cm lesion with close contact with the trachea originated from the muscular wall of the oesophagus

Oral and oesophageal cysts may cause respiratory distress or dysphagia. Retrosternal pain, haemoptysis and infection can occur in case of large cysts with rapid growth (Fig. 6) [6, 23].

**Fig. 7:**
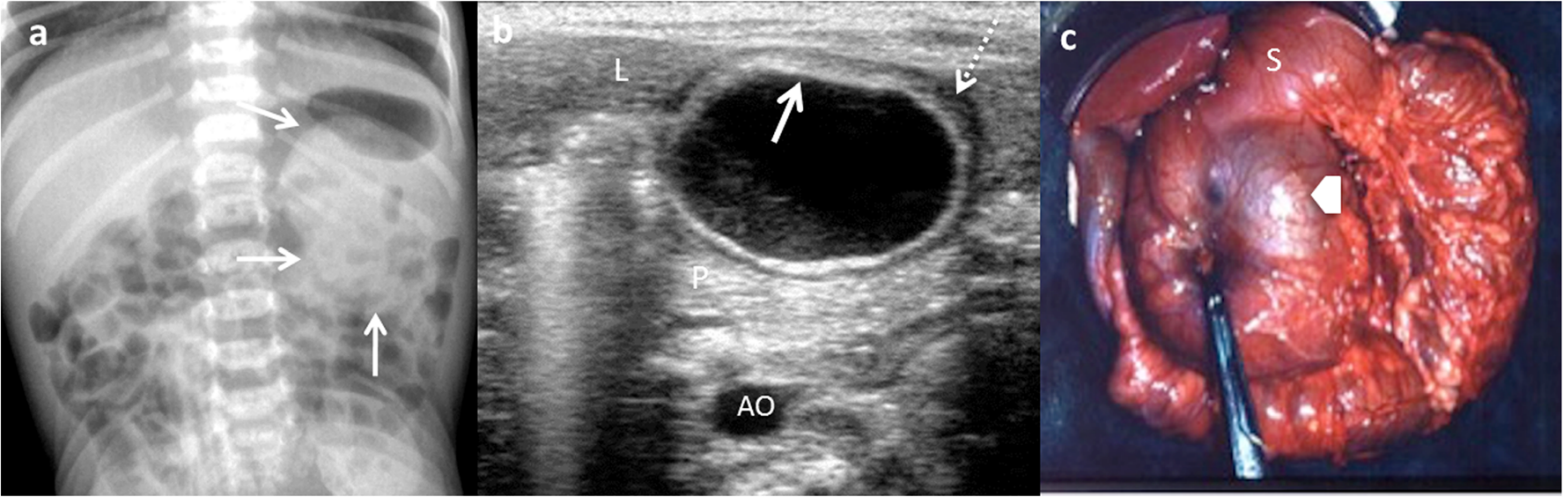
An 18-month-old girl with an abdominal mass in the physical exam is studied. **a** Abdominal X-ray: round, dense mass is discovered (*arrows*) in the left upper quadrant. **b** Transversal US view of the lesion: a cyst with the “double-wall” sign: the mucosa is hyperechoic (*arrow*) and the muscular layer is hypoechoic (*dashed-line arrow*). A gastric duplication cyst was suspected. *L* liver, *P* pancreas, *AO* aorta. **c** Laparotomy: antral duplication cyst was found (*arrow*). *S* stomach

Gastric and intestinal duplications may produce nausea, vomiting, abdominal distention or palpable abdominal mass (Fig. 7). Recurrent abdominal pain is one of the most frequent forms of presentation and is usually attributed to high pressure inside the duplication cyst because of the accumulation of secretions. Intussusception is another complication in which the cyst serves as a head point and pain, obstruction or bleeding are possible forms of manifestation. Extrinsic compression of the adjacent bowel is also possible, which causes obstruction. However, the most serious complications are produced if gastric mucosa is present within the cyst, leading to inflammation, bleeding, ulceration and even perforation [6, 9, 13, 14, 24, 25].

**Fig. 8:**
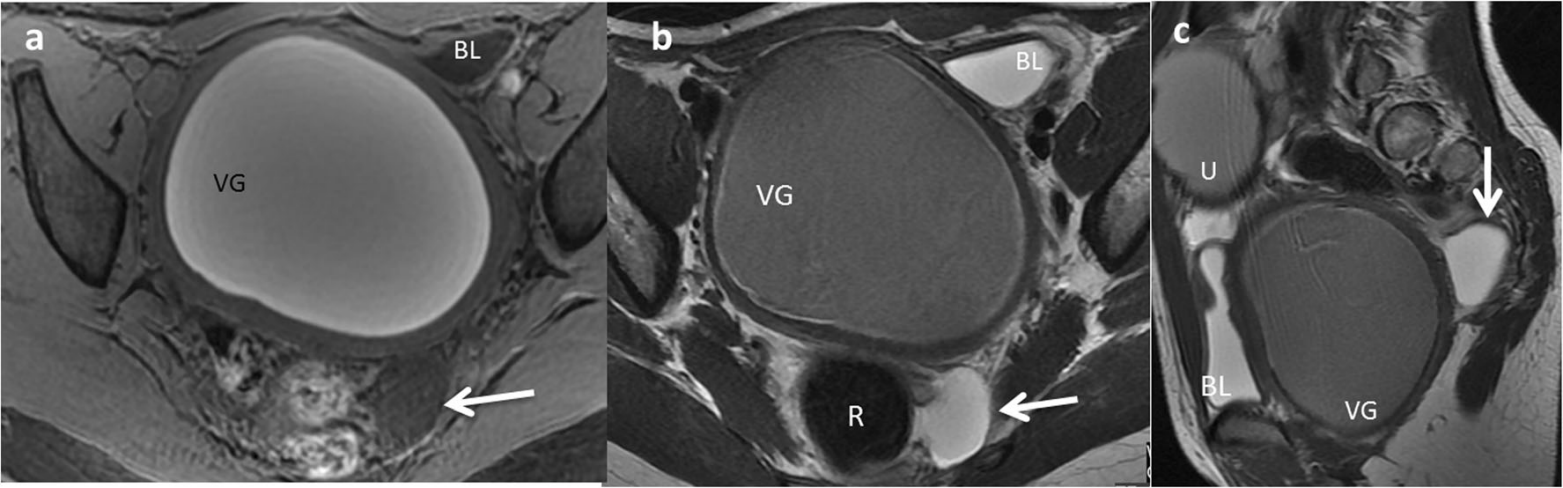
A 12-year-old girl with a gynaecological malformation and haematometrocolpos. **a** A hypointense lesion is seen (*white arrow*) next to the rectum in axial T1 weighted pelvic MR. **b** On T2-weighted pelvic MR, the cyst next to the left wall of the rectum is seen. **c** Sagittal plane of the T2 MRI showing the posterior location of the cyst (*arrow*). *VG* vagina, *BL* bladder, *U* uterus. Imaging findings of a rectal duplication cyst. Note the haemorrhagic content of the uterus and vagina because of haematometrocolpos

Nonetheless, EDCs can sometimes be detected incidentally (Fig. 8).

## Imaging findings

US is the imaging method of choice in the diagnosis of EDCs; only limited in the evaluation of oesophageal EDCs. Transoesophageal ultrasound might be useful but is not routinely used [26, 27].

Classical findings of uncomplicated EDC include: presence of a cyst in relation to the gut with double-wall or muscular rim sign (gut signature sign), which is caused by inner hyperechoic mucosa and outer hypoechoic smooth muscle layer (muscularis propia) (Figs. 1 and 7). However, the double-wall sign in other cystic lesions (mesenteric cyst, Meckel’s diverticulum or torsioned ovarian cyst) may be seen [1, 2, 11, 24, 28–30].

**Fig. 9:**
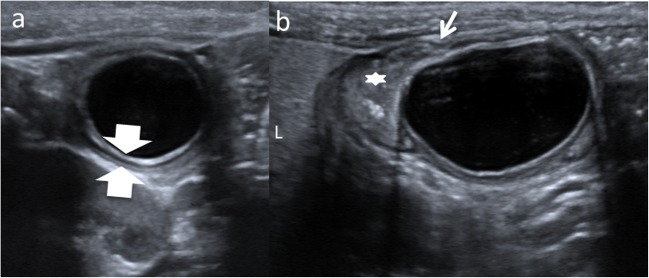
A 3-month-old boy with vomiting is admitted to the emergency room. **a** US shows a cystic round-shape lesion with the “five-layers sign” (between *arrows*). **b** The “Y sign” is seen (*long arrow*). *Star* ileum, *L* liver. Laparoscopic findings: a non-complicated ileal duplication cyst

New US signs are described according to the characteristics of the EDCs:

- As an EDC contains the same multi-layered wall architecture as the normal GT, the sign “five-layered cyst wall” is proposed. It corresponds to the innermost hyperechoic mucosa, hypoechoic muscularis mucosa, hyperechoic submucosa, hypoechoic muscularis propia and the outermost hyperechoic serosa. Identification of all five layers in a cyst is pathognomonic of EDC. However, this sign is difficult to demonstrate and needs expertise and high-resolution US (12–18 MHz) [27, 29, 30]. For this reason, the use of US linear probe is recommendable when the GT is examined.
- An EDC shares wall with the adjacent GT. Therefore, the diagnosis is carried out if it is possible to demonstrate the “Y” sonographic configuration of the muscle layer caused by the splitting of the shared muscularis propria between the cyst and the adjacent loop. This sign is not described for other abdominal cysts and reflects one of the histological characteristics of the EDCs (Fig. 9) [29, 31].

**Fig. 10:**
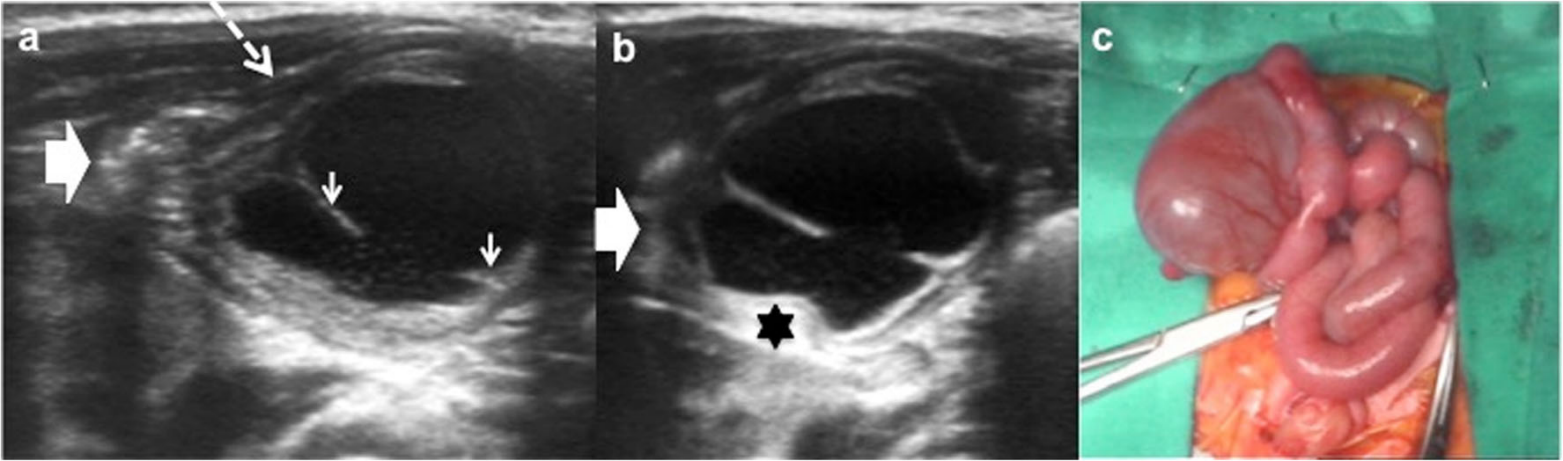
An 8-month-old boy with abdominal pain. **a** Longitudinal grey-scale US image showing a cystic lesion with an incomplete septum inside (*small white arrows*), next to the terminal ileum (*big arrow*). The “Y” sign is shown (*dashed-line arrow*). **b** US image obtained a few seconds later: peristalsis of the cyst causes small angulation of the contour and changes shape (*black star*). **c** Surgical findings: ileal duplication cyst

US is a dynamic study and allows to visualise the peristalsis of the cyst wall. It appears as a transient change of the shape and contour of the cyst because of a concentric contraction of the cyst wall (Fig. 10) when the transductor stays still on the cyst for a while [2, 27].

**Fig. 11:**
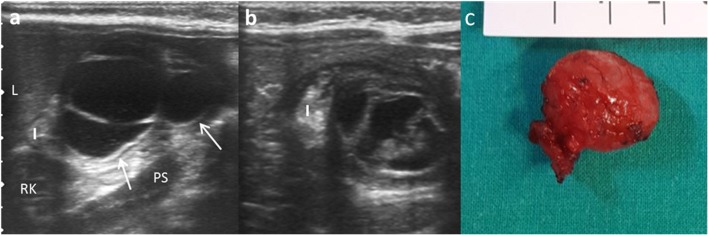
An 8-month-old boy with abdominal pain and abdominal mass on physical exam. **a** Longitudinal US view of a multiseptated cystic mass in the right flank with the “Y” sign (*white arrows*). *I* ileum, *L* liver, *Ps* psoas, *RK* right kidney. **b** Transversal US of the same mass demonstrates the relation with the ileal walls. **c** Surgical findings: ileal duplication cyst

Although EDCs are frequently anechoic or hypoechoic, mucinous material or septations can be present without being complicated (Fig. 11) [27].

**Fig. 12:**
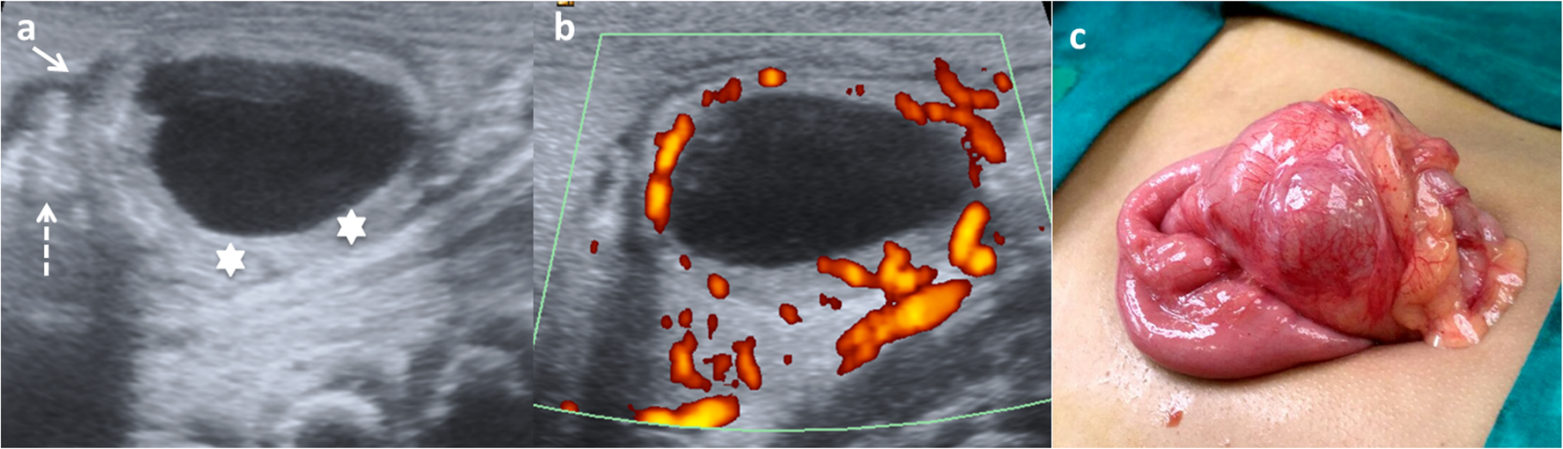
A 3-week-old term newborn with abdominal distention and gastric intolerance. **a** Longitudinal US view of the right low quadrant: thickened wall (*stars*) cystic lesion next to a bowel loop (*dashed-line arrow*). The “Y” sign between the bowel and the lesion (*arrow*). **b** Power Doppler US demonstrates the significant vascularisation in the cyst wall. **c** Surgical findings: a cystic tumour next to the ileocecal valve. Pathological findings: ileal duplication cyst with heterotopic pancreatic tissue

**Fig. 13:**
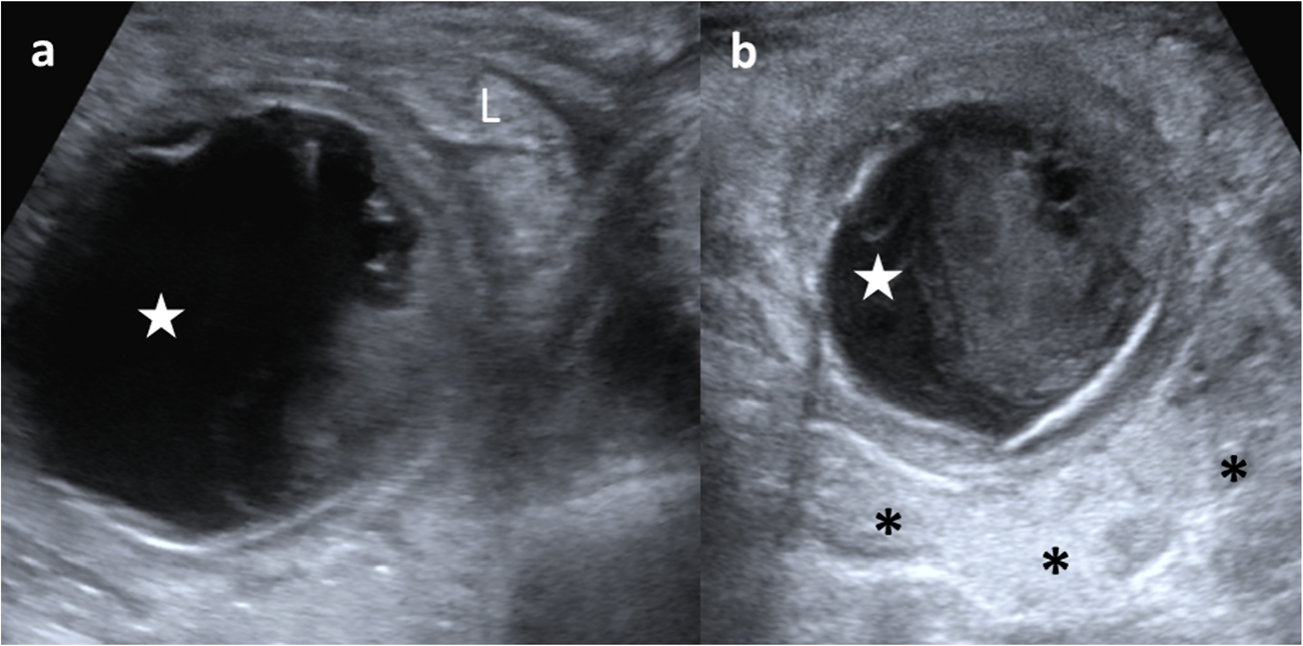
A 3-year-old boy with fever and abdominal pain is studied. **a** US shows a cystic mass (*star*) with internal debris and next to an ileal loop (*L*). **b** The lesion (*star*) is surrounded by echogenic mesenteric fat (*) as an inflammatory sign. Surgical findings: a 5-cm ileal complicated duplication cyst was found with gastric mucosa with haemorrhagic and ulcerated walls

**Fig. 14:**
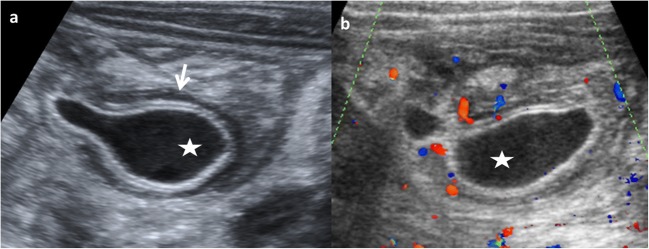
An 8-month-old boy baby with continuous crying is taken to the emergency department. Because of high suspicion of intussusception, a US exam is required. **a** Abdominal pear-shaped cystic lesion (*star*) in the left flank was found in a coronal US view. It shows a typical outer hypoechoic wall and inner hyperechoic layer, with hypoechoic content inside. **b** Doppler US showing light hyperaemia of the lesion wall that was also thickened. Surgical findings: complicated duplication cyst in the colonic splenic flexure

**Fig. 15:**
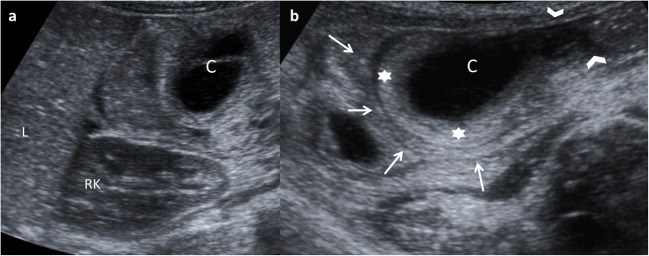
A 14-month-old girl with acute gastroenteritis and continuous crying. **a** Abdominal US shows an intestinal intussusception with a cyst (*C*) as the leading cause. *L* liver, *RK* right kidney. **b** A detailed US view: the intussuscipiens (*arrows*) and intussuscepted bowel (*arrowheads*) with the cyst inside (*C*) and the hyperechoic and thickened walls. Surgical findings: ileal duplication cyst as the cause of the intussusception

Complicated EDCs rarely present the classic five layers or double-wall sign. Ectopic rest of pancreatic tissue can produce enzymatic destruction of the mucosa with inflammation, as well as loss of the wall layers showing a hyperaemic thick wall. In such cases, the “Y configuration” sign helps in establishing the correct diagnosis of EDC (Fig. 12) [3, 29, 30]. If ectopic gastric mucosa produces haemorrhage and bleeding, fluid levels or echogenic debris can be seen. When infection occurs, ulceration of mucosa can appear, and internal debris may be seen. The transmural extension can produce important inflammatory changes in the surrounding mesentery fat (Figs. 13 and 14) [3, 7, 24]. Ileal EDC, near the ileocecal valve, can act as intussusception head, showing on US a cystic mass inside the intussusception requiring emergency treatment (Fig. 15) [2].

In case of atypical or isolated EDC, the pseudokidney sign is described when there is a complete loss of typical wall layers because of severe congestion, thus producing a thick hypoechoic rim with a hyperechoic central layer [32].

**Fig. 16:**
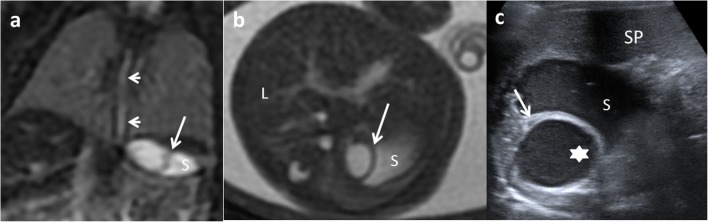
A 22-week-old fetus with an abdominal cyst seen on ultrasound is studied with MR. **a** Coronal fetal FIESTA MR: a cystic lesion is seen next to the stomach (*S*). Oesophageal lumen is seen (*arrows*). **b** FIESTA transversal MR: the hypointense wall of the suspected gastric duplication cyst (*arrow*). *L* liver, *S* stomach. **c** Postnatal abdominal US view: the lesion (*star*) imprinting the gastric wall (*arrow*). The content of the stomach is seen (*S*). *SP* spleen. Surgical findings: duplication cyst of the oesophageal-gastric transition

US prenatal diagnosis of EDCs includes the same signs as postnatal cyst: the double–wall sign and the presence of peristalsis. However, on the prenatal US, the “double wall” is not always seen or can be partial [10, 33, 34], and it may require the differential diagnosis with other cystic lesions such as mesenteric, omental, ovarian and choledochal cysts. If it is possible to demonstrate the presence of peristalsis in the cyst wall, an intestinal origin is probed. MR imaging is suggested to have a supplemental value in the assessment of fetal abdominal cysts (Fig. 16) [10, 35, 36].

**Fig. 17:**
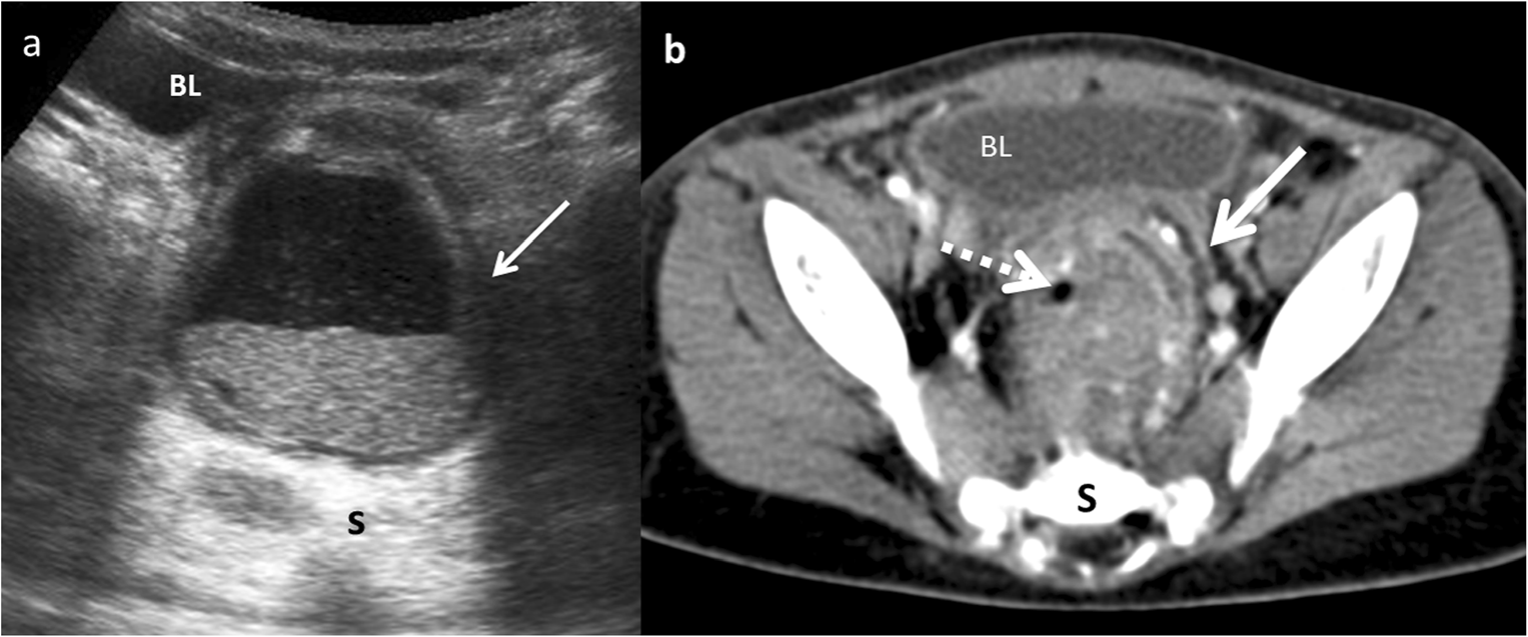
A 22-month-old boy with fever and abdominal pain. **a** Transversal US view of the pelvis shows a cystic mass between the bladder (*BL*) and the sacrum (*S*). The lesion presents anterior and left wall thickening (*arrow*) and contains a fluid-fluid level suggesting the presence of a complicated rectal mass. **b** Contrast-enhanced CT confirmed the presence of a complicated rectal cyst with remarkable inflammatory findings (*white arrow*) and gas (*dashed-line arrow*). Surgical findings: rectal duplication cyst with mucous and purulent content. Rectum muscular wall was oedematous and it was shared with the duplication cyst

CT is not typically performed for evaluation of EDCs due to radiation. CT may depict the location and extension of the cyst, as well as complications, the associated anomalies and anatomical relationship with surrounding structures. At CT, an EDC manifests as a cystic mass with a thin and slightly enhancing wall adjacent to the gastrointestinal wall. A high attenuation inside the cyst may be seen due to haemorrhage or proteinaceous material. A thick enhancing wall, air bubbles inside and cyst-surrounding inflammation may indicate an EDC complicated by infection (Fig. 17) [1, 11, 12, 15].

**Fig. 18:**
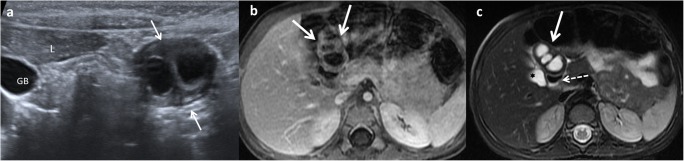
A 2-year-old girl with fever and abdominal pain. **a** Abdominal US view of a complex cystic lesion (*white arrows*) in the duodenal area, next to the liver (*L*). *GB* gallbladder. **b** Axial gadolinium-enhanced GRE T1 demonstrating the presence of a multilocular cystic mass (*white arrows*) with wall thickening. **c** Axial T2 MRI: the mass (*white arrow*) shows three cystic cavities inside, close to the anterior wall of the duodenum (*dashed-line arrow*) and lateral to the gallbladder (*star*). Surgical findings: multiple duodenal duplication cyst, without connection with the lumen and with ectopic gastric mucosa

Like CT, MR is not routinely used as a diagnostic method for EDCs, especially due to sedation requirement. On MR imaging, most duplications have low signal intensity on T1-weighed images and very high intensity on T2-weighted images (Fig. 18). Both CT and MR play a major role prior to surgery in establishing the relationship between the cyst and its adjacent structures [12], and in locations where US presents a limited use, particularly in oesophageal and rectal duplications [1, 2, 6, 12].

## Management

The main considerations in the management of EDCs are: the condition of the patient, the location of the cyst, whether it involves one or more anatomic locations, whether its structure is cystic or tubular, and if it is communicated with the true intestinal lumen.

With the widespread availability of antenatal diagnosis, EDCs are often diagnosed prenatally. The optimal time to perform the resection in children with antenatal diagnosis is not defined. These patients should undergo early investigation, followed by early resection even within the first 6 months of life [3, 37, 38].

Treatment of asymptomatic EDCs remains controversial. The clinical behaviour of EDCs is unpredictable. EDCs tend to increase in size gradually and can cause symptoms and important complications that might be fatal, such as obstruction, massive bleeding or even a potential risk for malignant transformation in the adulthood [13, 14, 17, 39].

Early excision is associated with less morbidity and a shorter length of stay compared to excision in symptomatic patients. There are significant post-operative morbidities after resection of complicated EDCs, compared with its elective surgery. Cyst excision alone could be considered, but if there is a communication, sometimes a resection of the adjacent bowel is necessary. It is important to ensure that the cyst is entirely resected because recurrence or malignant changes may occur [40].

Currently, minimally invasive surgery is becoming the elective approach, and most of the cysts can be resected successfully, either thoracoscopically or laparoscopically, as long as an exhaustive imaging diagnosis is available [41].

## Conclusions

EDCs are uncommon congenital abnormalities arising anywhere along the GT. Their clinical presentations vary according to the site of duplication; ileum appears as the most commonly involved. Nowadays, antenatal diagnosis is becoming more frequent. US is the method of choice to diagnose gastrointestinal EDCs. Although double-wall US sign in a cyst is the most typical for diagnosis of EDCs, the findings of the five layers sign or the “Y configuration” of the muscular layer are more specific features. Complicated cysts present atypical imaging findings. CT and MR imaging can be required in oesophageal or rectal EDCs for planning complicated surgical approach. Surgery is necessary because of the severe complications they can develop. The diagnosis is confirmed by histological examination.

## Abbreviations

EDC: Enteric duplication cyst
GT: Gastrointestinal tract
